# Meningeal Lymphatics Drives Macrophage Clearance via CCL2-CCR2 Axis After Cerebral Ischemia

**DOI:** 10.3390/cimb48030259

**Published:** 2026-02-28

**Authors:** Jing Wang, Yu Lei, Yongfeng Yang, Jin Wang

**Affiliations:** Department of Physiology and Pathophysiology, State Key Laboratory of Medical Neurobiology, School of Basic Medical Sciences, Fudan University, Shanghai 200030, China

**Keywords:** macrophage, meningeal lymphatic vessels, VEGF-C therapy, CCL2-CCR2 axis, ischemic stroke

## Abstract

The mechanisms underlying meningeal lymphatic vessel (mLV)-mediated immune cell clearance after stroke remain unclear. Using a mouse middle cerebral artery occlusion model, we performed single-cell RNA sequencing to analyze post-ischemic meningeal macrophages. In vitro co-culture and CCR2 inhibition (RS504393) validated the CCL2-CCR2 axis between lymphatic endothelial cells and macrophages. Macrophage trafficking to mLVs and cervical lymph nodes was assessed by Evans Blue tracing and F4/80 immunofluorescence. We utilized VEGF-C to enhance meningeal lymphatic vessel function and concomitantly evaluated neurological deficits, brain edema, and neuroinflammation. Ischemia expanded meningeal macrophages, whose crosstalk with lymphatic endothelial cells relied on CCL2-CCR2 axis. CCR2 inhibition impaired macrophage trafficking to mLVs and cervical lymph nodes, worsening edema, motor deficits, and inflammation, whereas VEGF-C enhanced mLV drainage and improved outcomes. We identify a novel mechanism where in mLVs recruit macrophages via CCL2 for perivascular clearance post-ischemia. Combining VEGF-C with modulation of the CCL2-CCR2 axis presents a promising synergistic therapeutic strategy for stroke.

## 1. Introduction

Ischemic stroke, a common occlusive condition affecting the cerebral blood vessels, continues to be a major disease causing death and long-term impairment [1]. After middle cerebral artery occlusion (MCAO) in mice, microglia located in the infarct core promptly trigger inflammatory cascades [2,3], driving blood–brain barrier (BBB) impairment, brain edema, neuronal death, and impaired tissue repair [4,5]. Crucially, current therapeutic interventions—intravenous thrombolysis and endovascular therapy—are restricted to narrow time windows (4.5–6 h post-onset) and fail to address ischemia–reperfusion injury, which exacerbates infarct expansion and neurological deficits [6,7]. These limitations underscore the urgent need to explore alternative pathogenic mechanisms beyond the parenchyma, particularly those involving neuroimmune crosstalk in the meningeal compartment.

Emerging evidence now positions the meninges as a dynamic immunological interface, hosting diverse immune cell populations that surveil the CNS and orchestrate responses to injury, infection, and degenerative processes [8,9,10,11]. This tri-layered membrane harbors diverse immune cells that surveil neural tissue and modulate disease progression. The meningeal immune niche comprises macrophages, dendritic cells, Mucosal-associated invariant T cells (MAIT), B cells, and innate lymphoid cells, distributed across the dura, arachnoid, and pia mater [8,9]. Unlike the parenchymal microglia, these cells interact directly with cerebrospinal fluid (CSF), peripheral blood, and skull bone marrow channels—forming a bidirectional immune–central nervous system (CNS) communication axis [10]. Dural macrophages patrol perivascular spaces, capturing blood-borne pathogens such as viruses and bacteria before CNS invasion [12]. Immature MHCII^hi^ macrophages around dural sinuses impair antiviral responses, facilitating viral penetration into the CNS [13]. Post-infection, meningeal macrophages are replaced by peripheral monocytes with reduced Toll-like receptors (TLRs), diminishing future defense capacity [12]. In tuberculous meningitis, macrophages act as “Trojan horses,” transporting bacteria across the blood–CSF barrier and evading antimicrobial drugs [14]. Meningeal immunity contributes to neuroinflammation and amyloid clearance. Aged meninges exhibit pro-inflammatory T-cell clones that infiltrate the parenchyma via CXCL16 signals from degenerating microglia, exacerbating neuronal damage [15]. After ischemic stroke, skull bone marrow-derived neutrophils rapidly infiltrate infarct sites via meningeal channels, releasing neutrophil extracellular traps (NETs) that amplify injury [10]. In addition, immune cells secrete factors such as antioxidants and tight-junction proteins critical for meningeal barrier integrity. For example, MAIT cells limit oxidative stress via selenoproteins, preventing barrier leakage and subsequent neuroinflammation [8]. During inflammation, meningeal endothelial and epithelial cells release CCL2 and CXCL12 chemokines to recruit monocytes and neutrophils [14,16]. Recent findings have recognized the meningeal lymphatic system as an essential drainage pathway independent of the BBB, which is mainly necessary for removing macromolecules and metabolic waste from the brain [17]. Given that meningeal lymphatic vessels (mLVs) transport immune cells from the CNS to peripheral lymph nodes, several studies have now investigated their role in neurological disorders characterized by neuroinflammation [18,19]. We therefore hypothesize that targeting meningeal immune pathways may unlock novel neuroprotective strategies for stroke management.

Meningeal lymphatic vessels are vital for the clearance and drainage of cerebrospinal fluid (CSF) that contains macromolecular waste as well as in establishing and maintaining immune surveillance in various diseases like Alzheimer’s disease and Parkinson’s disease [18,20,21,22]. A disruption in meningeal lymphatic drainage can worsen disease progression and cognitive decline, particularly in neurodegenerative and age-associated neurological disorders [22,23,24]. Research has also indicated that meningeal lymphatic vessels may aid in clearing erythrocytes from the brain following subarachnoid hemorrhage (SAH) [25]. When traumatic brain injury (TBI) occurs, a pathology of disruption of meningeal vascular integrity is often observed in patients [26]. And promotion of meningeal lymphatic drainage can decrease the severe outcomes of TBI [27]. Furthermore, investigations have explored the link between the lymphatic drainage system and immune, although the precise role of meningeal lymphatic vessels in the progression and aftermath of ischemic stroke remains a topic of debate [28,29]. Therefore, it is necessary to explore the interaction between meningeal lymphatic vessels and immune cells after stroke.

Here, we identify a CCL2–CCR2 axis as a key mediator bridging macrophage recruitment to mLVs and subsequent inflammation resolution after middle cerebral artery occlusion (MCAO). We demonstrate that meningeal lymphatic endothelial cells (mLECs) upregulate CCL2 expression in response to post-ischemic inflammation, and macrophages are recruited to perivascular meningeal lymphatic vessel (mLV) niches via CCR2-CCL2 chemotaxis. And this recruitment facilitates macrophage clearance through mLV-mediated drainage, reducing neuroinflammation and improving neurological outcomes. Therapeutic VEGF-C delivery amplifies mLV function, enhancing CCL2-dependent macrophage clearance and improving neurological outcomes. These findings position meningeal lymphatics not merely as passive drainage routes but as active immunomodulatory hubs that can be therapeutically harnessed to accelerate stroke recovery.

## 2. Materials and Methods

### 2.1. Animals and Cerebral Ischemia Model

#### 2.1.1. Mice

All mice with a C57BL/6 genetic background were bred and housed in a Specific Pathogen Free (SPF) environment at the Laboratory Animal Services Centre of Fudan University monitoring tests, were kept in a controlled environment with a temperature of 23 ± 1 °C and humidity of 50 ± 10%, on a 12 h light/dark cycle (lights on at 8:00), and had free access to normal rodent feed and sterile tap water. The injury and Sham groups were randomly allocated from a total of 120 male and female C57BL/6J mice weighing 22–25 g (approximately 10 weeks old). Wild-type C57BL/6 mice were purchased from The Jackson Laboratory (The Jackson Laboratory, Cat # 000664).

#### 2.1.2. Middle Cerebral Artery Occlusion

Focal cerebral ischemia was induced through transient middle cerebral artery occlusion (MCAO) as previously described [30]. Briefly, mice were anesthetized with 3% isoflurane followed by 1.0–1.5% isoflurane (R510-22-10, RWD Life Science, Shenzhen, China) via nose cone. A feedback-regulated heating pad was used to keep body temperature within the range of 37.0 ± 0.5 °C. Following a midline cervical incision, a silicone-coated monofilament suture (diameter selected by weight: 0.22 ± 0.01 mm, 25–30 g; 0.20 ± 0.01 mm, 20–25 g; 0.18 ± 0.01 mm, 15–20 g; Beijing Cinontech.) was introduced into the right common carotid artery (CCA) via arteriotomy and advanced into the internal carotid artery (ICA) to occlude the origin of the middle cerebral artery (MCA) for 30 min. Reperfusion was established by filament withdraw. Sham-operated mice received identical anesthesia, neck incision, vessel dissection, and suture placement around the ECA/ICA as MCAO mice, but without filament insertion or artery occlusion.

#### 2.1.3. Cerebral Blood Perfusion

Cerebral blood flow (CBF) dynamics were assessed via laser speckle contrast imaging (LSCI; PeriCam PSI System NR PSI-30059, Perimed AB, Stockholm, Sweden). Following a midline scalp incision under anesthesia with 3% isoflurane followed by 1.0–1.5% isoflurane, transcranial LSCI measurements were acquired through the intact skull. Cortical perfusion was quantified and expressed in arbitrary perfusion units (PU), as defined by the instrument software.

#### 2.1.4. TTC Staining

Infarct volume quantification followed established TTC protocols [31]. Briefly, 24 h post-reperfusion, coronal slices were incubated in 2% TTC solution at 37 °C for 20 min to undergo staining. After fixation in 4% PFA for 10 min, sections were imaged using a Leica stereomicroscope (M205 FA). Infarct areas were quantified relative to total section area using ImageJ 2.3.0. Infarct Volume Percentage = ∑Contralateral Hemisphere Volume/∑ [Contralateral Hemisphere Volume − Ipsilateral Non-Infarcted Volume] × 100%.

### 2.2. Neurobehavioral and Pathophysiological Assessments

#### 2.2.1. Brain Water Content Test

Brain edema was quantified using the wet–dry weight technique, as detailed in prior research [32]. At 24 h after transient middle cerebral artery occlusion (MCAO), mice were sacrificed under profound anesthesia induced by 2.5% tribromoethanol. The brains were promptly excised and weighed right away to determine the wet weight (WW). Subsequently, the tissues were dehydrated at 105 °C for 72 h to acquire the dry weight (DW). Brain water content (%) = (WW − DW)/WW × 100%.

#### 2.2.2. Wire Hanging Test

At 3 days post-MCAO surgery, a horizontal iron wire (1 mm in diameter, 55 cm in length) was stretched between two posts, positioned 50 cm above the ground. Mice were placed on the wire and required to suspend their body weight using their forelimbs. To avoid the use of all four paws, their hind limbs were gently restrained with adhesive tape. A soft pillow was placed under the mice to prevent injury in case of falls. The test duration was capped at 60 s. Prior to the formal testing, mice received three daily training sessions.

#### 2.2.3. Open Field Test

Behavioral testing was conducted in a white polypropylene open-field arena (40 × 40 × 40 cm) under 15-lux illumination. Mice were acclimated to the testing room for ≥3 h before trials to reduce stress and ensure stable behavioral performance. Before each test, the arena was thoroughly sanitized with 75% ethanol to eliminate residual olfactory cues, and software was calibrated and initialized for data acquisition (EthoVision XT 16.0). Each mouse was gently placed in the center of the arena with its head facing away from the experimenter to avoid directional bias. Locomotor activity, including total distance traveled, average velocity, and time spent in different zones, was continuously recorded for 15 min. Between individual subjects, the arena was re-sanitized with ethanol and fully dried to prevent cross-contamination of olfactory signals.

### 2.3. Whole-Mount Immunofluorescence Staining of Meninges

#### 2.3.1. Whole-Mount Immunostaining of Meninges

Following a 3-day reperfusion period, the mouse underwent perfusion with 0.1 M PBS, and the skull-adherent meninges were fixed in 4% paraformaldehyde (PFA) at 4 °C. After fixation, the meninges were washed three times with 0.1 M PBS for 20 min each. The skulls were decalcified in 0.5 M EDTA at 4 °C overnight, followed by washing with PBS. Following permeabilization with 0.3% Triton X-100 for 1 h, the tissues were incubated overnight at 4 °C with the following primary antibodies: rabbit anti-LYVE-1 (1:200 dilution; Cat# MAB2125, R&D Systems, Minneapolis, MN, USA.) and rat anti-F4/80 (1:200 dilution; Cat# ab6640, Abcam, Cambridge, UK). Then the skulls were washed three times with 0.3% Triton X-100 for 10 min each. The tissues were then incubated for 1 h at room temperature with the corresponding secondary antibodies: Alexa Fluor^®^488 donkey anti-rabbit IgG (Cat#A21206; Invitrogen, Carlsbad, CA, USA, 1:1000,) and Alexa Fluor™ 594 donkey anti-rat IgG (H + L) (Cat#A21209; Invitrogen, Carlsbad, CA, USA, 1:1000). Following staining, the skulls were washed three times with 0.3% Triton X-100 for 10 min each. DAPI was applied for 10 min at room temperature, and the skulls were subsequently washed three times with 0.3% Triton X-100 for 10 min each. The meninges were subsequently detached from the skulls, spread onto glass slides, and observed under a Leica SP8 confocal microscope.

#### 2.3.2. Immunofluorescence Staining

Brains were fixed with 4% paraformaldehyde and embedded in Tissue-Tek^®^ O.C.T (Sakura Finetek USA, Torrance, CA, USA). Compound for frozen sections and sectioned at 30 μm intervals for brain. The slides were blocked with 0.3% Triton X-100 containing 1% BSA for 1 h, and then incubated with the following primary antibodies at 4 °C overnight, rabbit anti-NeuN (Cat#ab177487; Abcam, Cambridge, UK, 1:200) and rabbit anti-CD45 (Cat#A23503; Abclonal, Wuhan, China, 1:200). After primary antibody incubation, the slides were washed three times with PBS for 10 min per wash. They were then incubated with secondary antibodies Alexa Fluor^®^488 donkey anti-rabbit IgG (Cat#A21206; Invitrogen, Carlsbad, CA, USA 1:1000), Alexa FluorTM594 donkey anti-rabbit IgG(H + L) (Cat#A21207; Invitrogen, Carlsbad, CA, USA 1:1000) or Alexa Fluor^®^647 donkey anti-rabbit IgG (Cat#A31573; Invitrogen, Carlsbad, CA, USA 1:1000) for 1 h and DAPI for 10 min at room temperature with rotation. Following secondary antibodies’ incubation, the slides were washed three times for 10 min per wash. Images were captured using a fluorescent microscope and the fluorescent density was quantified by ImageJ 2.3.0 software (National Institutes of Health, Bethesda, MD, USA).

### 2.4. Bioinformatics Analysis

RNA-seq data of post-stroke meninge samples (GSE254550) were retrieved from the NCBI GEO database. Raw sequencing data were quality-checked using FastQC, followed by trimming of low-quality reads and adapters with Trimmomatic to obtain clean reads. And gene expression levels were quantified with FeatureCounts, generating read count matrices which were then normalized. Differential expression analysis was performed using DESeq2, with differentially expressed genes (DEGs) identified by the criteria of adjusted *p*-value < 0.05 and |log2FoldChange| > 1. Gene Ontology (GO) enrichment analysis of DEGs was conducted using the ClusterProfiler package in R. CellChat analysis was performed to explore cell–cell communication networks based on the normalized gene expression matrix.

### 2.5. Molecular Biology Analyses

#### 2.5.1. RNA Extraction and Quantitative Real-Time PCR (qPCR)

The mRNA expression levels of target genes were analyzed using qRT–PCR. The meningeal tissues were homogenized in 1 mL of TRIzol reagent and incubated at room temperature for 5 min to ensure complete dissociation of nucleoprotein complexes. For phase separation, 0.2 mL of chloroform was added per 1 mL of TRIzol used. The mixture was vigorously shaken by hand for 15 s and then incubated at room temperature for 2–3 min. The samples were subsequently centrifuged at 12,000× *g* for 15 min at 4 °C. The upper aqueous phase containing total RNA was carefully transferred to a new RNase-free tube, avoiding contamination from the interphase or organic phase. RNA was precipitated from the aqueous phase by adding 0.5 mL of isopropanol (100%) per 1 mL of the initial TRIzol volume. The mixture was incubated at room temperature for 10 min and then centrifuged at 12,000× *g* for 10 min at 4 °C to pellet the RNA. After discarding the supernatant, the RNA pellet was washed with 1 mL of 75% ethanol. The pellet was briefly vortexed to dislodge and centrifuged at 7500× *g* for 5 min at 4 °C. The ethanol wash was removed, and the RNA pellet was air-dried for 5–10 min at room temperature. It is critical to avoid over-drying the pellet, as this can drastically reduce RNA solubility. The purified RNA pellet was dissolved in 100 μL of RNase-free water, and RNA concentration and purity were verified using a NanoDrop spectrophotometer. Complementary DNA (cDNA) synthesis was carried out using the HiScript II One Step RT-PCR Kit (Vazyme) following the manufacturer’s instructions. qRT–PCR was conducted on a Baiyuan Gene ASA-900 real-time PCR system (EZB) with ChamQ^TM^ qRT-PCR SYBR Green master mix (EZB). The thermal cycling program consisted of an initial denaturation at 95 °C for 30 s, followed by 40 cycles of denaturation at 95 °C for 10 s and annealing/extension at 60 °C for 30 s. The 2ΔΔCt method was employed to determine relative gene expression levels, with GAPDH serving as the internal control. The primers are listed in Table A1.

#### 2.5.2. Western Blot Analysis

Total cellular proteins were extracted using 100 μL RIPA lysis buffer supplemented with 1% (*v*/*v*) protease/phosphatase inhibitors (1:100) on ice for 30 min. The lysates were centrifuged at 12,000× *g* for 15 min at 4 °C. The resulting clear supernatants were collected, and protein concentrations were determined using a BCA protein assay kit. For electrophoresis, equal amounts of protein samples were loaded onto 12% SDS-PAGE gels. Electrophoresis was performed initially at 70 V for 45 min, followed by 130 V for 90 min to separate the proteins. The separated proteins were then transferred onto PVDF membranes at a constant current of 300 mA for 60 min. The membranes were blocked with 5% (*w*/*v*) non-fat milk for 1 h at room temperature and subsequently incubated overnight at 4 °C with gentle shaking in primary antibody solution containing rabbit anti-CCR2 (Cat#A2385, Abclonal, Wuhan, Hubei, China, 1:1000). After incubation, the membranes were washed three times with TBST for 5 min each to remove unbound primary antibody. They were then incubated with HRP-conjugated goat anti-rabbit IgG secondary antibody (Cat#SA00001-2, Proteintech, Wuhan, Hubei, China, 1:2500) for 1 h at room temperature, followed by three washes with TBST for 5 min each. For blot reprobing, the membranes were incubated in mild antibody stripping buffer (Cat# PS107S, Yamei, Shanghai, China) for 20 min at room temperature and washed three times with TBST for 5 min each before being re-blocked and reprobed using the standard protocol described above. Protein bands were visualized by the Tanon 5200 multi-imaging system. The gray values of the brands were calculated by ImageJ 2.3.0 software (National Institutes of Health, Bethesda, MD, USA).

### 2.6. Cell Culture

Mouse lymphatic endothelial cells (mLECs) were cultured in complete growth medium, high glucose-DMEM medium supplemented with 10% fetal bovine serum (FBS) and 1% penicillin-streptomycin. To simulate ischemic conditions in vitro, the LECs were subjected to oxygen-glucose deprivation/reperfusion (OGD/R). The cells were then placed in a hypoxic chamber equilibrated with a gas mixture containing 1% O_2_, 5% CO_2_, and 94% N_2_ for 3 h to induce OGD. Following the OGD phase, the glucose-free medium was collected. This conditioned medium (CM) from the OGD period (OGD-CM) could be replaced with fresh standard medium for 24 h to simulate reperfusion, and that subsequent supernatant (OGD/R-CM) might also be collected. Conditioned medium was collected from LECs (NC-CM) in normal oxygen chamber containing 20% O_2_, 5% CO_2_, and 75% N_2_ maintained under normal culture conditions. The collected CM was centrifuged to remove any cellular debris. LEC-medium with or without CCR2 antagonist (Cat#HY-15418, Med Chem Express, Monmouth Junction, NJ, USA,10 μM) was then applied to RAW 264.7 murine macrophage cells. For scratch assay, confluent macrophage monolayers were scratched with 200-μL pipette tips. After PBS washing, cells were treated with LEC-conditioned medium ± CCR2 antagonist (10 μM). Wound closure was monitored by microscope for 24 h. Wound closure was calculated as: [1 − (wound area at 24 h/wound area at 0 h)] × 100.

### 2.7. Flow Cytometry

Two meninges were collected and pooled into a single tube per sample. The meninges were chopped into small fragments and digested into single-cell suspensions using papain (Cat#76216, Sigma-Aldrich, St. Louis, MO, USA; 0.25% in EBSS), collagen II (Cat#C6885, Sigma-Aldrich St. Louis, MO, USA; 450 U/mL in HBSS with 90 mM Ca^2+^), and dispase II (Cat#D4693-1G, Sigma-Aldrich, St. Louis, MO, USA; 1 U/mL in EBSS). For each digestion solution, DNase I (Cat#DN25, Sigma-Aldrich, St. Louis, MO, USA; 100 U/mL) was added. The digestion was terminated by adding cold HBSS. The cell suspension was then filtered through a 70 μm cell strainer. The resulting single-cell suspensions were subjected to centrifugation at 300× *g* for 10 min at room temperature. The pellet was washed twice with PBS. Cell pellets were resuspended in 50 μL of blocking buffer, which contained 1 × PBS, 0.5% bovine serum albumin, and 2 mM ethylenediaminetetraacetic acid tetrasodium salt dihydrate. Subsequently, cells were stained on ice for 30 min with the following antibodies: eFluor450 anti-mouse CD45 (Cat#12-9459-41, Invitrogen, Carlsbad, CA, USA, 1:100) and BV650 anti-mouse CD11b (Cat# 101259, Biolegend, San Diego, CA, USA, 1:100). For single-stained control tubes, two separate tubes of single-cell suspension (at the same concentration as experimental samples) were prepared for fluorescence compensation calibration to eliminate spectral overlap between channels. One tube was stained only with eFluor450 anti-mouse CD45 (Cat#12-9459-41, Invitrogen, Carlsbad, CA, USA, 1:100), and the other tube was stained only with BV650 anti-mouse CD11b (Cat# 101259, Biolegend, San Diego, CA, USA, 1:100). All staining conditions were identical to those of the experimental samples. For the isotype controls tube, equal volume of single-cell suspension was stained with isotype-matched IgG control antibodies corresponding to the fluorochromes of CD45 and CD11b antibodies under the same incubation conditions to define background fluorescence and exclude non-specific antibody binding. For positive controls, peripheral blood was collected from healthy C57BL/6 mice via the orbital venous plexus into centrifuge tubes containing heparin sodium for anticoagulation to validate antibody reactivity, instrument sensitivity and the reliability of the CD45^+^CD11b^+^ gating strategy. In total, 1 mL of 1× RBC Lysis Buffer was added to peripheral blood, gently inverted to mix, and incubated at room temperature for 5–10 min. The suspension was directly centrifuged at 500× *g* for 5 min. The supernatant was discarded, and 1 mL of PBS containing 2–10% FBS was added to resuspend the pellet, followed by centrifugation at 500× *g* for 5 min for washing. After discarding the supernatant, the cell pellet was resuspended in 100 μL of medium with 10% FBS, and eFluor450 anti-mouse CD45 (Cat#12-9459-41, Invitrogen, Carlsbad, CA, USA, 1:100) and BV650 anti-mouse CD11b (Cat# 101259, Biolegend, San Diego, CA, USA, 1:100) were added. The suspension was incubated in the dark at 4 °C for 20 min. Following incubation, all experimental and control cells were centrifuged at 300× *g* for 5 min and resuspended in 500 μL of PBS for immediate analysis. Flow cytometry was performed using NovoCyte 3000 flow cytometer (Agilent Technologies, Santa Clara, CA, USA).

### 2.8. Pharmacological Intervention

#### 2.8.1. Injection into Cisterna Magna

Mice were anesthetized with 3% isoflurane followed by 1.0–1.5% isoflurane (RWD Life Science, Shenzhen, Guangzhou, China) via nose cone. A feedback-regulated heating pad was used to keep body temperature within the range of 37.0 ± 0.5 °C. The dorsal neck skin was shaved, followed by disinfection with iodine and 75% ethanol, and penicillin eye ointment was applied to protect the eyes. After securing the mouse’s head, the skin and muscle layers were dissected to expose the cisterna magna. The target solution was delivered into the cisterna magna using a microliter syringe (Hamilton 10 μL, Hamilton Company, Reno, NV, USA). Upon completion of the injection, the needle was retained in position for 5 min to avoid cerebrospinal fluid reflux. The skin incision was then closed with nylon sutures, and the mice were placed on a heating pad to recover until they regained full consciousness.

To assess Evans Blue (EB) (Cat#314-13-6, Med Chem Express, Monmouth Junction, NJ, USA) extravasation and perform quantification, a 5% EB solution was administered into cisterna magna. Specifically, 5 μL of the EB solution was infused into the target region at a constant flow rate of 1 μL/min. After the administration of EB, fluorescent signal of both sides of the cervical lymph nodes (CLNs) was captured by a fluorescence imaging system of VISQUE In vivo Smart-LF (Vieworks, Anyang, Republic of Korea) after a 1 h interval.

Recombinant VEGF-C protein at concentration of 0.025 μg/μL for 5 μL (rVEGF, Cat# C546, Novoprotein, Suzhou, Jiangsu, China) was administered into the cisterna magna at an infusion rate of 1 μL per minute. Subsequently, staining was performed as outlined in the prior literature [18].

#### 2.8.2. Administration of CCR2 Antagonist

CCR2 was pharmacologically inhibited by intraperitoneal injection of RS504393 (Cat#HY-15418, Med Chem Express, Monmouth Junction, NJ, USA) with 5 mg/kg. The first dose was administered 6 h post-reperfusion, followed by daily injections for 3 days [33]. Vehicle control mice received equivalent volumes of solvent.

### 2.9. Statistical Analysis

Statistical analyses were performed using GraphPad Prism 9.0 software. All data are presented as the mean ± Standard Error of the Mean (SEM) unless otherwise specified. Differences between two groups were analyzed using the unpaired Student’s *t*-test, while comparisons among multiple groups were conducted via one-way analysis of variance (ANOVA) followed by Tukey’s post hoc test. Normality was evaluated using the Shapiro–Wilk test (α = 0.05) prior to parametric analysis. A *p*-value less than 0.05 was considered statistically significant.

## 3. Results

### 3.1. Ischemic Injury Induces Neuroinflammation via Immune Cell Activation and Inflammatory Factor Accumulation, Contributing to Neuronal Damage

**Figure 1:**
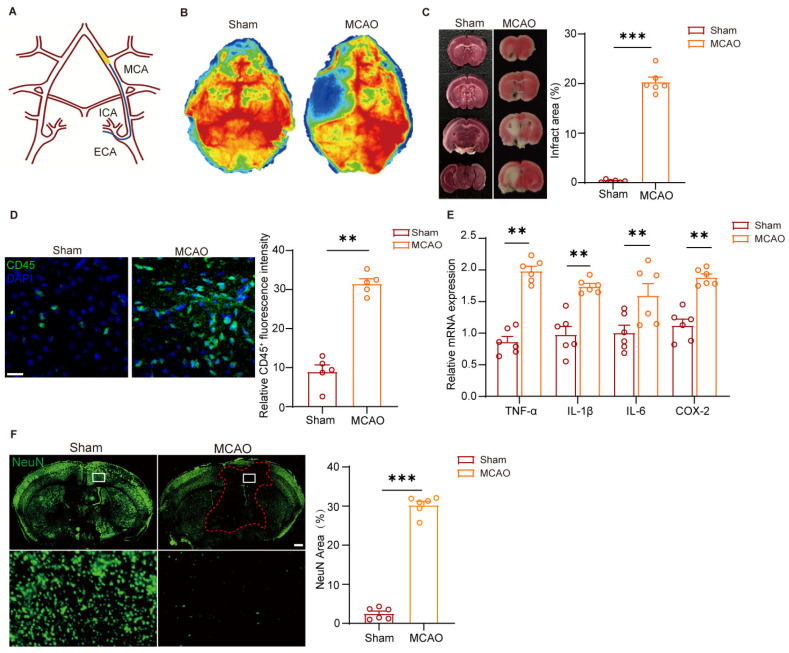
Ischemic injury induces neuroinflammation and neuronal damage. (**A**) The diagram of cerebral ischemic injury by guide wire. MCA: middle cerebral artery, ICA: internal carotid artery, ECA: external carotid artery. (**B**) Representative images of cerebral blood flow in the Sham and MCAO mice. (**C**) Representative images and quantification of TTC (2,3,5-Chlorotriphenyltetrazolium) staining in the Sham and MCAO injured *C57BL/6* mice. Sham group: *n* = 6; MCAO group: *n* = 6. The red color indicates viable tissue with functional mitochondrial enzyme activity, while the white region represents the infarct zone. (**D**) Representative images and quantification of CD45^+^ Immunofluorescence in the brain parenchymal section of indicated mice. Sham group: *n* = 6; MCAO group: *n* = 6. Scale bar = 50 μm, magnification, ×20. (**E**) TNF-α, IL-1β, IL-6 and COX-2 were measured by quantitative polymerase chain reaction in brain parenchyma of Sham and MCAO *C57BL/6* mice. *n* = 6. (**F**) Representative images and quantification of NeuN immunostaining in the brain parenchymal section from Sham and MCAO injured *C57BL/6* mice. Sham group: *n* = 6; MCAO group: *n* = 6. Scale bar: 100 μm, magnification, *×*10. Data in (**C**,**D**,**F**) are presented as mean ± SEM and an unpaired *t* test was performed to calculate significance between two groups. Data in (**E**) are presented as mean ± SEM, and two-way ANOVA was performed to calculate significance between two groups. ** *p* < 0.01, *** *p* < 0.0001.

To investigate the pathological role of meningeal lymphatics and associated immune responses in cerebral ischemia, we developed a mouse model of middle cerebral artery occlusion (MCAO) (Figure 1A) to verify the successful establishment of the ischemic brain injury model. After 1 h of occlusion, the absence of blood supply on the left lateral brain was observed through Laser Speckle Contrast Imaging (LSCI) (Figure 1B). Compared with the Sham mice, cerebral infarction of MCAO group was confirmed by TTC (2,3,5-triphenyltetrazolium chloride) staining (Figure 1C). We found that increased CD45^+^ leukocytes accumulated a lot in the infarct zone of MCAO mice (Figure 1D). Consistent with the established role of inflammation in ischemic injury, we found that MCAO triggered a pronounced neuroinflammatory response, which was characterized by elevated levels of inflammatory mediators such as TNF-α, IL-1β, IL-6, and COX-2 (Figure 1E). And we found that the neural network units were impaired, while further documented by a dramatical decrease in NeuN^+^ cells in the infarct zone area after reperfusion (Figure 1F). These findings demonstrated that ischemic injury induces neuroinflammation through immune cell activation and inflammatory factor accumulation.

### 3.2. Lymphatic-Mediated Macrophage Recruitment Fuels Inflammatory Exacerbation After Stroke

**Figure 2:**
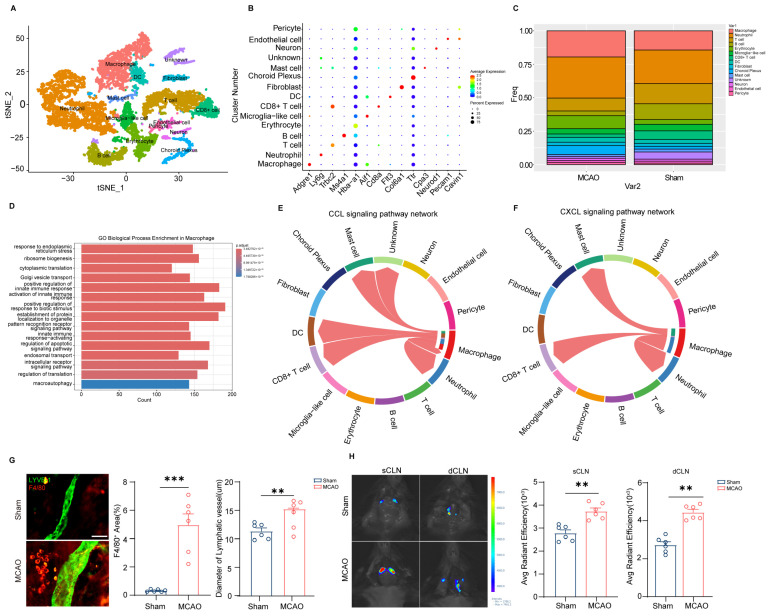
Lymphatic-mediated macrophage recruitment fuels inflammatory exacerbation after stroke. (**A**) tSNE dimensionality reduction analysis displayed clusters of meninges in Sham and MCAO *C57BL/6* mice. (**B**) The dotplot of top marker gene in all cell types. (**C**) Frequency distribution of all clusters in Sham and MCAO *C57BL/6* mice. (**D**) GO Biological Process enrichment analysis of macrophages. (**E**) Cell–cell communication analysis reveals CCL signaling pathway networks coordinated by macrophages. (**F**) Cell–cell communication analysis reveals CXCL signaling pathway networks coordinated by macrophages. (**G**) Representative images of F4/80 and LYVE-1 immunofluorescence, with quantification of F4/80 + Immunofluorescence and lymphatic diameter of the meninges in the indicated mice. Scale bar: 50 μm, magnification, ×20, Sham group: *n* = 6; MCAO group: *n* = 6. (**H**) Representative images of Evans blue in superficial cervical lymph nodes (sCLNs) and deep cervical lymph nodes (dCLNs) from Sham and MCAO *C57BL/6* mice, along with quantification of Evans blue are presented. Sham group: *n* = 6; MCAO group: *n* = 6. Data in (**G**,**H**) are presented as mean ± SEM and an unpaired *t* test is performed to calculate significance between two groups. ** *p* < 0.01, *** *p* < 0.0001.

Single-cell RNA-seq data from GSE254550 were processed using R 4.5.1. Following quality control (mitochondrial genes < 5%, 500 < nFeature_RNA < 3500), t-SNE embedding of 11,796 high-quality cells revealed 30 transcriptionally distinct clusters (Figure 2A). Cluster identities were annotated via canonical markers Macrophage (*Adgre1*, *Clec4a1*), Neutrophil (*Ly6g*, *Cd177*), T cell (*Cd3d*, *Trbc2*), B cells (*Ms4a1*, *Cd79a*), Erythrocyte (*Hba-a1*, *Hba-a2*), Microglia-like cell (*Aif1*, *Hebx*), CD8^+^ T cell (*Cd8a*, *Cd3d*), Dendritic cell (DC) (*Flt3*, *Ciita*), Fibroblast (*Col6a1*, *Cdh11*), Choroid Plexus (*Ttr*, *Folr1*), Mast cell (*Cpa3*, *Fcer1a*), Neuron (*Neurod1*, *Chgb*), Endothelial cell (*Kdr*, *Pecam1*), and Pericyte (*Cavin1*, *Rgs5*) (Figure 2B). Frequency analysis revealed a dramatic expansion of meningeal macrophages following ischemic injury macrophages, which comprised 14.34% of total meningeal cells in Sham controls, but surged to 20.57% post-MCAO (Figure 2C). The shift represented the most pronounced change among all immune subsets, implicating macrophages as key responders to cerebral ischemia. GO biological process enrichment analysis of macrophage-specific genes demonstrated a significant enrichment of terms related to immune signaling. Notably, pathways such as the ‘positive regulation of innate immune response’ and ‘innate immune response-activating signaling pathway’ were prominently enriched (Figure 2D), linking the expansion of these macrophage populations to the exacerbation of post-stroke inflammation. Cellchat analysis exhibited that macrophages communicate with immune cells, such as neutrophils, mast cells, Dendritic cells (DCs) and CD8^+^ T cells via the CCL and CXCL chemokine signaling networks, thereby coordinating and amplifying the overall inflammatory response (Figure 2E,F). Further immunofluorescence analysis revealed an accumulation of F4/80^+^ macrophages within the perivascular regions of LYVE1^+^ meningeal lymphatic vessels following MCAO (Figure 2G). Additionally, we observed augmented meningeal lymphatic drainage, evidenced by an increased Evans blue concentration in the superficial and deep cervical lymph nodes (sCLN and dCLN) and vessel diameter of meningeal lymphatics. This enhanced drainage is indicative of an accelerated clearance mechanism for the recruited macrophages post-MCAO (Figure 2G,H).

### 3.3. Macrophage Migration to the Meningeal Lymphatic Vessel Relies on the CCL2-CCR2 Signaling Pathway

**Figure 3:**
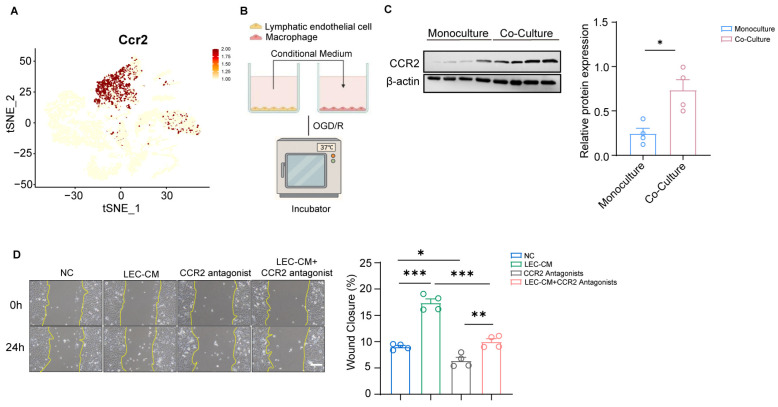
Macrophage migration to the meningeal lymphatic vessel relies on the CCL2-CCR2 signaling pathway. (**A**) Single-cell transcriptomic visualization (t-SNE) of *Ccr2* gene expression in macrophages. (**B**) Schematic of the indirect co-culture system modeling post-stroke. Conditioned medium from lymphatic endothelial cells subjected to oxygen-glucose deprivation/recovery (OGD/R) was collected and applied to macrophages to model the paracrine effects of meningeal lymphatic-derived factors following cerebral ischemia. (**C**) Representative Western blot images and corresponding quantitative analysis of CCR2 expression in macrophages. NC-CM group: *n* = 4, OGD/R-CM group, *n* = 4. NC-CM: Conditioned medium of lymphatic endothelial cell from normal oxygen, OGD/R-CM: Conditioned medium of lymphatic endothelial cell from the oxygen-glucose deprivation/recovery. (**D**) Representative images of macrophage scratch analysis and quantitation of wound closure in NC-CM (Conditioned medium of lymphatic endothelial cell from normal oxygen), OGD/R-CM (Conditioned medium of lymphatic endothelial cell from the oxygen-glucose deprivation/recovery), CCR2 antagonist, OGD/R-CM + CCR2 antagonist. NC-CM group: *n* = 4, OGD/R-CM group, *n* = 4; CCR2 antagonist group: *n* = 4; OGD/R-CM+ CCR2 antagonist group *n* = 4. Scale bar = 50 μm, magnification, ×20. Data in (**C**) were presented as mean ± SEM and an unpaired *t* test was performed to calculate significance between two groups. Data in (**D**) are presented as mean ± SEM and ordinary one-way ANOVA was performed to calculate significance between four groups. * *p* < 0.05, ** *p* < 0.01, *** *p* < 0.0001.

Single-cell transcriptomic analysis revealed that the gene encoding the CCL2 receptor, Ccr2, was specifically and highly expressed in macrophages (Figure 3A). Therefore, we hypothesized that CCL2 secreted by meningeal lymphatic vessels acts on macrophages to induce their recruitment towards these vessels. To test this, we established an in vitro model in which macrophages were treated with conditioned medium collected from LEC cultures with or without oxygen-glucose deprivation/reperfusion (OGD/R) (NC-CM, OGD/R-CM) (Figure 3A). Macrophages treated with OGD/R-CM showed elevated CCR2 protein expression by Western blot compared to NC-CM (Figure 3B). To validate our hypothesis that macrophages migrate toward meningeal lymphatic vessels via the CCL2-CCR2 axis after MCAO, we employed a CCR2 antagonist RS504393 to test this mechanism. Notably, treatment with the CCR2 antagonist alone significantly suppressed macrophage migration, as evidenced by a marked reduction in wound closure compared to the NC-CM group, confirming the critical role of CCR2 in mediating basal macrophage motility (Figure 3D). Conversely, OGDR-CM markedly enhanced macrophage migration velocity and wound closure capacity. Critically, this pro-migratory effect was abolished when CCR2 antagonist was added to OGD/R-CM (Figure 3D). These findings indicate that CCL2 derived from lymphatic endothelial cells (LECs) drives macrophage migration via CCR2 signaling, which facilitates macrophage perivascular accumulation.

### 3.4. CCR2 Inhibition Impairs Macrophage Clearance by MENINGEAL Lymphatics in Stroke Recovery

**Figure 4:**
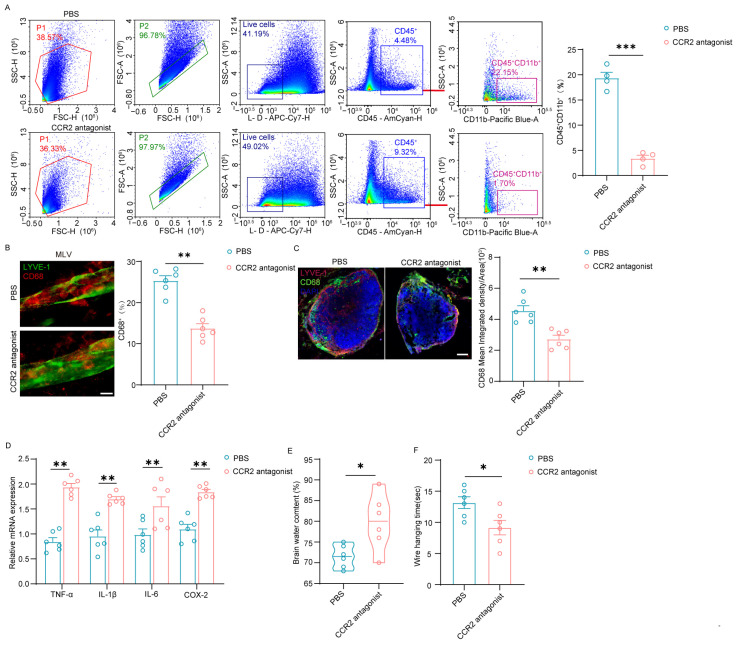
Intervention in CCR2 impairs meningeal lymphatic-mediated macrophage clearance and recovery post-ischemic injury. (**A**) Representative flow cytometry plots and quantitation of CD45^+^CD11b^+^ in the Sham and MCAO *C57/BL* mice with treated PBS or CCR2 antagonist RS504393. PBS group: *n* = 4, CCR2 antagonist group: *n* = 4. (**B**) Representative immunofluorescence staining images of F4/80 and LYVE-1 in the meninges of indicated mice, with corresponding quantitation. PBS group: *n* = 6, CCR2 antagonist group: *n* = 6. Scale bar: 20 μm, magnification, ×40. (**C**) Representative immunofluorescence staining images of F4/80 and LYVE-1 in sCLNs and dCLNs, with corresponding quantitation. PBS group: *n* = 6, CCR2 antagonist group: *n* = 6. Scale bar = 200 μm, magnification, ×20. (**D**) TNF-α, IL-1β, IL-6 and COX-2 were measured by quantitative polymerase chain reaction in brain parenchyma of indicated mice. *n* = 6. (**E**) Quantification analysis of brain water content in the indicated mice. *n* = 6. (**F**) Quantification analysis of hanging time (in seconds) by wire-hanging test in the indicated mice. *n* = 6. Data in (**A**–**C**,**E**,**F**) are presented as mean ± SEM and an unpaired two-tailed *t* test was performed to calculate significance between two groups. Data in (**D**) are presented as mean ± SEM and two-way ANOVA was performed to calculate significance between two groups. * *p* < 0.05, ** *p* < 0.01, *** *p* < 0.0001.

To evaluate the therapeutic potential of targeting of the CCL2–CCR2 axis, CCR2 antagonist RS504393 was applied to cerebral injured mice. Flow cytometry revealed that administration of the CCR2 antagonist RS504393 post-MCAO significantly reduced the proportion of CD45^+^CD11b^+^ macrophages within the meninge compared to PBS-treated controls (Figure S1 and Figure 4A). F4/80^+^ cells decreased significantly near meningeal lymphatic vessels compared to PBS group via immunofluorescence (Figure 4B). Decreased F4/80^+^ intensity in superficial (sCLN) and deep (dCLN) cervical lymph nodes, confirming decreased macrophage recruitment and activity after treatment with CCR2 antagonist RS504393 post-MCAO, which demonstrated that CCR2 antagonists block the drainage of macrophages by meningeal lymphatic vessels (Figure 4C). Real-time quantitative PCR demonstrated that CCR2 inhibition robustly contributed to the post-ischemic upregulation of key pro-inflammatory cytokines, including TNF-α, Il-1β, Il-6 and COX-2 (Figure 4D). This augmented neuroinflammatory cascade was associated with a significant increased brain water content (Figure 4E). Mice treated with the CCR2 antagonist exhibited significantly shorter latency to fall on wire hanging test, indicating poor motor coordination and functional recovery after MCAO (Figure 4F).

### 3.5. VEGF-C Improves Neurological Outcomes Post-Stroke via Promoting Drainage Function of Meningeal Lymphatics

**Figure 5:**
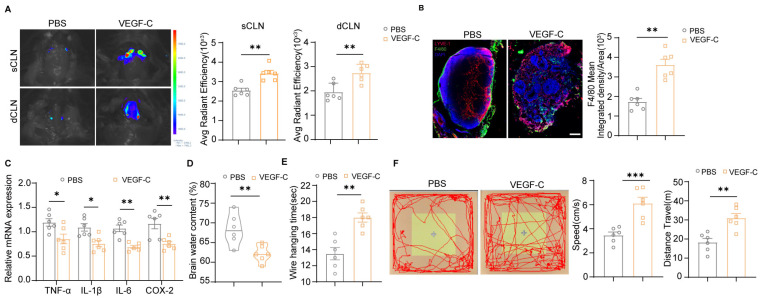
VEGF-C promotes macrophage clearance via meningeal lymphatics to enhance post-stroke recovery. (**A**) Representative images of Evans blue in sCLNs and dCLNs from MCAO injured PBS and VEGF-C-treated mice and quantification of EB concentration in CLN. PBS group: *n* = 6, VEGF-C group: *n* = 6. (**B**) Representative whole-mount immunostaining images of LYVE-1 and F4/80 in dCLNs from PBS- or VEGF-C-treated MCAO mice, with corresponding quantification of F4/80 fluorescence intensity. Scale bar = 200 μm, magnification, ×20. PBS group: *n* = 6, VEGF-C group: *n* = 6. (**C**) TNF-α, IL-1β, IL-6 and COX-2 were measured by quantitative polymerase chain reaction of brain parenchyma of MCAO injured PBS and VEGF-C-treated mice. PBS group: *n* = 6, VEGF-C group: *n* = 6. (**D**) Quantification analysis of brain water content in the indicated mice. PBS group: *n* = 6, VEGF-C group: *n* = 6. (**E**) Quantification analysis of hanging time (in seconds) by wire-hanging test in the indicated mice. PBS group: *n* = 6, VEGF-C group: *n* = 6. (**F**) Representative open field test (OFT) results and quantitation of movement speed and total traveled distance in the indicated mouse groups. Yellow square: central zone; brown area surrounding the central zone: peripheral zone, PBS group: *n* = 6, VEGF-C group: *n* = 6. Data in (**A**,**B**,**D**–**F**) are presented as mean ± SEM and an unpaired *t* test was performed to calculate significance between two groups. Data in (**C**) are presented as mean ± SEM and two-way ANOVA was performed to calculate significance between two groups. * *p* < 0.05, ** *p* < 0.01, *** *p* < 0.0001.

Given the shared lymphatic impairment in ischemic stroke, we hypothesized that augmenting meningeal lymphatic function via VEGF-C could contribute to macrophage migration following MCAO-induced injury. To test this, recombinant VEGF-C was administered to MCAO mice as a gain-of-function intervention. It was noted that there was a rise in the Evans Blue concentration in both sCLN and dCLN of MCAO mice treated with VEGF-C (Figure 5A). Additionally, VEGF-C treatment significantly increased macrophage accumulation in dCLN (Figure 5B), consistent with reduced expression of inflammatory chemokines, including TNF-α, Il-1β, Il-6 and COX-2 (Figure 5C). This demonstrates that VEGF-C enhances meningeal lymphatic drainage of macrophages to peripheral lymph nodes (LNs), effectively alleviating neuroinflammation (Figure 5B,C). VEGF-C-treated mice exhibited significantly attenuated cerebral edema and improved motor function, evidenced by reduced brain water content, prolonged wire hanging latency, increased ambulatory speed and total movement distance in open field (Figure 5D,F). These results suggested that VEGF-C treatment can enhance lymphatic drainage function after MCAO.

## 4. Discussion

As we all know, ischemic stroke commonly poses a huge threat to human health [34]. Brain ischemia triggers a sequence of events involving an inflammatory response, cerebral edema, and the accumulation of toxic substances [5,35], while restoring brain homeostasis can enhance neurological function. Recent research has highlighted the significance of lymphatic drainage systems in the pathology of neurodegenerative diseases, neuroinflammatory conditions, CNS injuries, and other neurological disorders [20,25,27,36]. Meningeal lymphatics have emerged as a pathway for the removal of abnormal proteins like Aβ and tau [18,37]. Degression of meningeal lymphatic function can impair the efficiency of clearance and lead to cognitive decline [20]. Studies on virus infections and autoimmune diseases have emphasized the connection between the brain-to-CLN pathway and inflammatory responses, influencing disease progression [38]. In an experiment involving an encephalomyelitis (EAE) mouse model of multiple sclerosis, the elimination of meningeal lymphatics using Visudyne delayed the progression of EAE in mice [39]. Crucially, impaired meningeal lymphatic function is linked to exacerbated pathology and cognitive decline, highlighting its role as a vital drainage and immunomodulatory pathway.

Recent studies reveal that meningeal lymphatic vessels (mLVs) play pivotal roles in immune cell clearance and inflammation regulation post-stroke [25,40]. However, the specific molecular mechanisms by which mLVs coordinate immune cell clearance remain unknown. Our study unveils a novel and active immune resolution pathway mediated by mLVs following ischemic stroke. Meningeal lymphatic vessels (mLVs) actively recruit macrophages via CCL2-CCR2 signaling, facilitating their perivascular clearance to mitigate neuroinflammation (Figure 3 and Figure 4). This mechanism fundamentally reshapes our understanding of mLVs—from passive drainage conduits to dynamic immunomodulatory hubs that orchestrate macrophage efflux after ischemic injury. Research has shown that meningeal lymphatic vessels can secrete chemokines to recruit immune cells [14,16]. While CCL2 is known to drive peripheral macrophage infiltration into the infarct core, we demonstrate its novel role in guiding macrophages to perivascular mLV niches for clearance. Disrupting this CCL2–CCR2 axis using the antagonist RS504393 significantly reduced macrophage clearance around mLVs and concurrently affected lymphatic drainage function (Figure 4). In summary, pharmacological blockade of CCR2 signaling exacerbated the expression of pro-inflammatory mediators, increased cerebral edema, and impaired functional recovery in a mouse model of ischemic stroke (Figure 4). This direct correlation links impaired macrophage-lymphatic trafficking to worsened post-stroke outcomes, firmly establishing mLVs as active immunomodulatory hubs essential for post-stroke inflammation resolution.

Importantly, our findings illuminate a profound bidirectional relationship between meningeal immunity and lymphatic function. Beyond their recruitment to the lymphatics, the macrophages accumulating perivascularly themselves emerge as potent signaling centers within the post-ischemic meningeal environment (Figure 2). CellChat analysis predicts that these macrophages are key senders of chemokine signals (CCL, CXCL) to other immune cells, suggesting they initiate a feedforward inflammatory cascade (Figure 2). This positions the perivascular mLV niche not just as a site of clearance, but as a critical “immune–lymphatic interface” within the meninges. Supporting this, in vitro co-culture experiments revealed that CCL2 derived from LECs enhance macrophage accumulation, an effect that is abolished by CCR2 inhibition, indicating that macrophages actively remodel the lymphatic vessels themselves (Figure 3C). Macrophages function as a pivotal communication hub in the post-stroke brain by orchestrating immune responses via CCL and CXCL chemokine signaling networks (Figure 2E,F). These findings demonstrated that macrophages were specifically activated and function as the central drivers of the inflammatory response following MCAO. Thus, the interaction between mLVs and macrophages represents a dynamic and reciprocal crosstalk fundamental to regulating meningeal immune responses after stroke; mLVs recruit macrophages for clearance, while the recruited macrophages actively modulate both the local inflammatory milieu and the lymphatic structures facilitating their own efflux.

The crucial role of the VEGFC-VEGFR3 signaling pathway in lymphangiogenesis, essential for the growth and development of meningeal lymphatic vessels in maintaining brain homeostasis is well established [41,42,43]. Dysfunction in the meningeal lymphatic system exacerbates neurological pathologies by impairing waste clearance and immune cell trafficking [44,45]. We have seen that infarct aggregation, neurological deficits and poor prognosis while the meningeal lymphatic defect mice (vegfr3 ^−/−^ suffer from ischemic brain injury [46]. Given that delivery of VEGF-C can contribute to meningeal lymphatic endothelial cell (mLEC) proliferation for improving the inflammation and cognitive dysfunction of Traumatic brain injury (TBI) [26,27], as a result, restoring meningeal lymphatic drainage has emerged as a promising therapeutic strategy across neurological disorders [41]. Given the shared lymphatic impairment in ischemic stroke, we hypothesized that augmenting meningeal lymphatic function via VEGF-C could contribute to macrophage migration following MCAO-induced injury. Our results suggested that VEGF-C treatment can enhance lymphatic drainage function after MCAO. VEGF-C therapy significantly improved stroke outcomes by enhancing meningeal lymphatic clearance. This augmentation of macrophage clearance effectively mitigated neuroinflammation and alleviated both cerebral edema and motor deficits (Figure 5). Importantly, our data designate VEGF-C and CCR2 as complementary therapeutic levers. While VEGF-C improved outcomes by boosting mLV-mediated efflux (Figure 5C,E,F), CCR2 blockade achieved comparable neuroprotection via the distinct mechanism of inhibiting macrophage influx.

Additionally, our study still has some limitations. Whether macrophage clearance occurs via trans-lymphatic transport or local apoptosis requires intravital imaging, we need to confirm CCL2–CCR2 axis activity in post-mortem stroke meninges using spatial transcriptomics. We propose a paradigm shift in stroke immunology that the meningeal lymphatic system is not merely a drain but a therapeutically exploitable gatekeeper that coordinates macrophage clearance via CCL2–CCR2 signaling. Augmenting this pathway via VEGF-C or modulating immune trafficking via CCR2 blockade presents new avenues to break the vicious cycle of post-stroke neuroinflammation. Future work should translate these mechanisms to human cerebrovascular disease and combinatorial immunotherapies.

## Data Availability

The data supporting this study’s findings are openly available in online repositories. Repository names and associated accession numbers are detailed below: https://www.ncbi.nlm.nih.gov/ (accessed on 14 January 2026) for GSE254550.

## Supplementary Materials

The following supporting information can be downloaded at: https://www.mdpi.com/article/10.3390/cimb48030259/s1.

## Abbreviations

The following abbreviations are used in this manuscript:

BBB	Blood–brain barrier
CCL2	CC chemokine ligand 2
CCR2	CC chemokine receptor 2
CLNs	Cervical lymph nodes
CM	Conditional medium
CNS	Central nervous system
CSF	Cerebrospinal fluid
dCLNs	Deep cervical lymph nodes
EAE	Encephalomyelitis
EB	Evans Blue
i.c.m	Inject into the cisterna magna
ICH	Intracerebral Hemorrhage
LECs	Lymphatic endothelial cells
LSCI	Laser Speckle Contrast Imaging
LYVE-1	Lymphatic vessel endothelial hyaluronan receptor-1
MAIT	Mucosal-associated invariant T
MCAO	Middle cerebral artery occlusion
mLECs	meningeal lymphatic endothelial cells
mLV	Meningeal lymphatic vessels
NETs	Neutrophil extracellular traps
OGD/R	Oxygen-glucose deprivation/recovery
rVEGF-C	Recombinant vascular endothelial growth factor-c
SAH	Subarachnoid Hemorrhage
sCLNs	Superficial cervical lymph nodes
TBI	Traumatic brain injury
TLRs	Toll-like receptors
TTC	2,3,5-triphenyltetrazolium chloride

## Appendix A

**Table A1:** The primers for Quantitative real-time PCR.

Gene	Primers
TNF-α	Forward: CAGGCGGTGCCTATGTCTC Reverse: CGATCACCCCGAAGTTCAGTAG
IL-1β	Forward: TTCAGGCAGGCAGTATCACTC Reverse: GAAGGTCCACGGGAAAGACAC
IL-6	Forward: TCTATACCACTTCACAAGTCGGA Reverse: GAATTGCCATTGCACAACTCTTT
COX-2	Forward: TGCACTATGGTTACAAAAGCTGG Reverse: TCAGGAAGCTCCTTATTTCCCTT
GAPDH	Forward: AGGTCGGTGTGAACGGATTTG Reverse: GGGGTCGTTGATGGCAACA
