# Supplementary material for: Meningeal Lymphatics Drives Macrophage Clearance via CCL2-CCR2 Axis After Cerebral Ischemia
Source: Curr Issues Mol Biol. 2026 Feb 28;48(3):259. doi: 10.3390/cimb48030259 (PMC13024750; doi:10.3390/cimb48030259)
Supplement: Supplementary file 1 [file cimb-48-00259-s001.zip › cimb-4128307-supplementary.pdf]

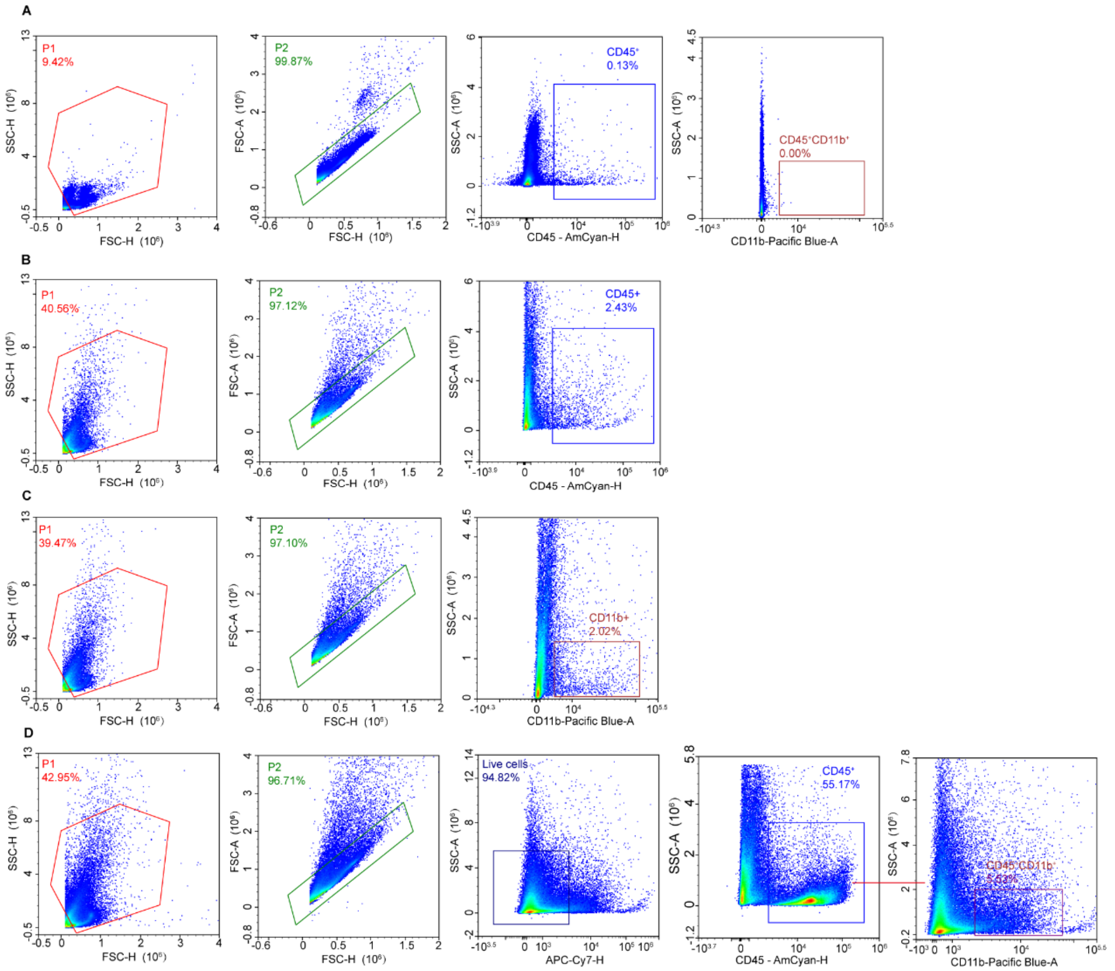

Figure S1. Gating strategy and validation controls for flow cytometric analysis of CD45⁺CD11b⁺ cells in mouse meninges. (A) Representative flow cytometry plots of meningeal single-cell suspensions stained with fluorochrome-matched isotype IgG control antibodies. (B) Representative flow cytometry plots of meningeal single-cell suspensions stained only with eFluor450 anti-mouse CD45. (C) Representative flow cytometry plots of meningeal single-cell suspensions stained only with BV650 anti-mouse CD11b. (D) Representative flow cytometry plot of positive control peripheral blood mononuclear cells (PBMCs) isolated from healthy C57BL/6 mice and stained with the same eFluor450 anti-CD45 and BV650 anti-CD11b antibodies as the experimental samples.
